# Variations in processes for guideline adaptation: a qualitative study of World Health Organization staff experiences in implementing guidelines

**DOI:** 10.1186/s12889-020-09812-0

**Published:** 2020-11-23

**Authors:** Zhicheng Wang, Quinn Grundy, Lisa Parker, Lisa Bero

**Affiliations:** 1 Charles Perkins Centre, Faculty of Medicine and Health, The University of Sydney School of Pharmacy, Sydney, Australia; 2 Lawrence S. Bloomberg Faculty of Nursing, University of Toronto, Toronto, Canada; 3 Colorado School of Public Health and Center for Bioethics and Humanities, University of Colorado School of Medicine, Aurora, USA

**Keywords:** WHO, Guidelines, Global health, Adaptation, Implementation, Research utilisation

## Abstract

**Background:**

The World Health Organisation (WHO) publishes a large number of clinical practice and public health guidelines to promote evidence-based practice across the world. Due to the variety of health system capacities and contextual issues in different regions and countries, adapting the recommendations in the guidelines to the local situation is vital for the success of their implementation. We aim to understand the range of experiences with guideline adaptation from the perspectives of those working in WHO regional and country offices. Our findings will inform development of guidance on how to improve adaptability of WHO guidelines.

**Methods:**

A grounded theory-informed, qualitative study was carried out between March 2018 and December 2018. Seventeen semi-structured interviews were conducted with participants who included WHO guideline developers and staff in the headquarters, regional and country offices recruited from a sample of published WHO guidelines. Participants were eligible for recruitment if they had recent experience in clinical practice or public health guideline implementation. Deidentified transcripts of these interview were analysed through three cycles of coding.

**Results:**

We categorised the adaptation processes described by the participants into two dominant models along a spectrum of guideline adaptation processes. First, the Copy or Customise Model is a pragmatic approach of either copying or customising WHO guidelines to suit local needs. This is done by local health authorities and/or clinicians directly through consultations with WHO staff. Selections and adjustments of guideline recommendations are made according to what the implementers deemed important, feasible and applicable through the consensus discussions. Second, the Capacity Building Model focuses on WHO building local capacity in evidence synthesis methods and adaptation frameworks to support local development of a national guideline informed by international guidelines.

**Conclusions:**

In comparing and contrasting these two models of guideline adaptation, we outline the different kinds of support from WHO that may be necessary to improve the effectiveness and efficiency of the respective models. We also suggest clarifications in the descriptions of the process of guideline adaptation in WHO and academic literature, to help guideline adaptors and implementers decide on the appropriate course of action according to their specific circumstances.

**Ethics:**

This project was conducted with ethics approval from The University of Sydney (Project number: 2017/723) and WHO (Protocol ID: 00001).

**Supplementary Information:**

**Supplementary information** accompanies this paper at 10.1186/s12889-020-09812-0.

## Background

The World Health Organization (WHO) is a major developer of both clinical practice and public health guidelines [1]. Since 2008, they have published more than 250 guidelines with rigorous methods of development to ensure their recommendations are evidence-based [1]. WHO is also at the forefront of taking into account issues related to transparency, independence, gender, equity, values and local contexts. These evidence-based guidelines aim to improve clinical and public health outcomes by helping health professionals practise in the most effective manner, as well as assisting policy-makers in designing optimal public health programs [2].

### Guideline implementation

Guidelines are only effective if they are adequately and appropriately disseminated and implemented. Enormous amounts of human and fiscal resources are poured into the development of WHO guidelines, but a recent study found the implementation plans included in WHO guidelines are often very brief and lack evidence for their effectiveness [3]. Studies have also found that the target audiences’ adherence to and uptake of guidelines are negatively affected by guidelines without adequate implementation plans [4, 5]. Without the proper implementation of guidelines by their intended users, the resources expended in the development of these guidelines by the health organisations will be wasted.

WHO works with six regional offices that are responsible for the countries in their respective regions [6]. Since the WHO guidelines are intended to be used in many different contexts and cultures, factors specific to these contexts need to be considered when implementation is being planned. The context in which WHO guidelines are implemented can be at different geographical levels: regional (e.g. Europe, South-East Asia or Western Pacific) or country. The context involves factors related to the local epidemiology, health system capacity and existing policies. Factors such as the local health culture and health system capacity need to be assessed by guideline implementers. The implementers also need to decide whether a particular WHO guideline is appropriate for their context and whether adaptation of the guideline is required.

### Adaptation as a part of guideline implementation

Guideline adaptation is a key part of the implementation process. It involves modifying a guideline so that it is suited to the regional or country context in terms of taking local human, resource and health system capacity into account as well as accommodating local values and preferences. Without adapting a guideline to suit the context of implementation, the end-users may find the guideline inappropriate for their setting [7]. The WHO Handbook for Guideline Development recommends the G-I-N process [8]. The Guidelines International Network (G-I-N) explains guideline adaptation as.

“the systematic approach to the endorsement and/or modification of a guideline(s) produced in one cultural and organisational setting for application in a different context. Adaptation may be used as an alternative to de novo guideline development, e.g., for customising (an) existing guideline(s) to suit the local context.” [9]

G-I-N recommends the ADAPTE framework, which describes a systematic way of developing a contextually specific guideline using available published evidence-based guidelines [10]. This process can be summarised as, first, forming a local guideline adaptation team (e.g. at a regional or country level). This team defines the local health questions that need to be answered and systematically searches available published guidelines to seek recommendations on the topic. This team then evaluates the available guidelines and contextualises the recommendations for their local situation. The adapted guidelines is then put through external review by target users, endorsement bodies and/or source guideline developers before being implemented [10, 11].

The ADAPTE framework is one of many that describe the process of guideline adaptation [11, 12]. These frameworks have evolved over the years to include different source materials for adaptation and have different recommendations for the adaptation process [13–15]. These frameworks are implemented at the local level and expect a level of methodological expertise in adaptation methods from the local guideline adaptation group. They are also quite resource intensive, in terms of human resources, money and time [11]. Apart from this high-level guidance, there is a lack of information in most WHO guidelines to effectively guide the local health workers to modify a specific guideline to their local context [3].

Recently WHO has made efforts to develop evidence-based guidelines that take into account a number of criteria in addition to evidence. These considerations include global ethical issues but also local values, preferences and capabilities. These factors are listed in the WHO-INTEGRATE evidence-to-decision framework: balance of health benefits and harms, human rights and sociocultural acceptability, health equity, equality and non-discrimination, societal implications, financial and economic considerations, and feasibility and health system considerations [16]. Guideline developers are expected to include and record their decision making process in light of these considerations in the guideline development process. It is hoped that having the developers consider these factors will result in guidelines that are more acceptable to local implementers. The documentation of the decision making processes will facilitate identification of contextual factors by local implementers that were considered by the guideline development group when making recommendations. Making these factors transparent enables guideline implementers to better assess the fit of the recommendations for their local context. Thus, some of the burden of guideline adaptation is shifted from the local implementers to guideline developers.

There have been few studies analysing the implementation of WHO guidelines. Most are individual case studies of implementation involving measuring uptake of particular WHO guideline recommendations (e.g. hand washing, trauma treatment, managing severely malnourished hospitalized children [17–19]). Collectively, these types of studies have tended to quantitatively explore the “success” of guideline implementation through measuring awareness of guideline recommendations [20], uptake of recommendations [21], and/or compliance to the guidelines as a whole [22]. None has focused on WHO officers’ experiences with adapting the guidelines to suit a range of local health contexts. We sought to qualitatively explore whether and how WHO guidelines are adapted during their implementation. Qualitative methods allow us to analyse the range of experiences in-depth to inform future guideline developers about common issues faced by the end-users and support effective guideline implementation.

### Research question

This paper describes the findings of part of a larger project exploring WHO guidelines adaptation and implementation [23]. While the previous paper described the multiple roles that WHO guidelines have throughout different regional and country offices, this paper will discuss differences in the ways WHO guidelines are adapted into local policies and practices. The aim of this paper is to understand the range of experiences with guideline adaptation from the perspectives of those working in the context of WHO regional and country offices. Our findings will inform development of guidance on how to improve adaptability of WHO guidelines.

## Methods

### Methodology and rationale

We conducted a qualitative study which involved semi-structured interviews with WHO guideline developers, staff and local implementers. This study used grounded theory methodology [24, 25], which aims to build an understanding (“theory”) about the process of guideline implementation directly from the data collected [24]. For the data to closely represent the participants’ experiences, we were open to factors that were not known in advance and used a systematic yet flexible approach to capture participants’ views, experiences and actions.

### Ethics approval

Ethics approval for this project was obtained from the Human Ethics Committee at The University of Sydney (Project number: 2017/723) and the Research Ethics Review Committee (WHO ERC) at WHO (Protocol ID: 00001).

### Sampling and eligibility

We recruited from the population of WHO staff who had recent experience in clinical practice or public health guideline implementation or had interactions with those who had implemented guidelines, identifying recent WHO guidelines (2007–2017) using their database (available on WHO website [1]). WHO guideline implementers are policy makers and health system advisers who implement the recommendations in the guidelines and drive change in national health policy and clinical practice. They are often WHO regional and country office staff who work closely with national health authorities and partners to achieve this goal. The extent of their training and experience in specific implementation and adaptation methods greatly vary. The methods they use would depend on their geographic location and specific guidelines they are implementing. We purposively recruited for participant variation in: guideline topic and department experience, WHO office level (e.g. Headquarters [HQ], Regional and Country offices) and geographic location, reasoning that this would provide us with the range of implementation experiences. We initially recruited from 15 WHO guidelines covering a variety of topics and WHO departments. Snowball sampling was then used to recruit local implementers who had worked with the first round of participants. We continued recruiting until we were confident that we had spoken with participants across a range of experiences and locations, and that only similar concerns continued to emerge (thematic saturation) We used Morse’s definition of saturation; “themes are considered saturated when data from several participants have essential characteristics in common [26].

### Data collection

ZW conducted the interviews between March 2018 and December 2018. He obtained informed consent from each participant. The interviews were audio recorded and the recording transcribed by a professional transcriptionist. To identify salient themes that arose and to encourage reflexivity, ZW made field notes before, during, and after the interviews [27].

The interview explored in detail the process of guideline implementation and adaptation, order of procedure (i.e. who initiated the process), barriers and facilitators, and the participants’ opinions about the process. The interview guide is available in Additional file 1 [23].

### Data analysis

The transcripts were deidentified using pseudonyms and removing participants’ department and location (the quotations presented in this manuscript are also under pseudonyms). Data analysis began simultaneously with data collection. Through this cycle, we explored ideas and issues that emerged through the analysis of early transcripts in subsequent interviews [24]. Early transcripts were read repeatedly to identify salient concepts which were used to build an initial coding tree. Examples of initial codes included, “WHO guidelines”- > “Guideline adaptation”-> “Adaptation projects in different countries”-> “Mish-mashing guidelines from different sources”. ZW wrote memos during the initial read to capture emerging ideas and patterns. An example of an early idea and pattern that emerged included “Definitions of guideline adaptation in different regions”.. Using the initial coding tree, ZW coded all transcripts in NVivo [28]. We iteratively revised and refined the coding tree through this process; examples of codes at this stage of analysis were “Engaging the national guidelines group,” and “Opinion leaders swaying the guideline”. ZW then grouped related codes regarding the systems of guideline adaptation into themes and conducted a third round of coding to group portions of transcripts into higher level categories. Key categories included “Implementation and policy change”, and “operationalisation of guidelines”.

Memos were a key tool in the data analysis process, which were used to explicate the categories, compare categories across participants and contexts, and integrate raw data into the emerging analysis [22]. We selected illustrative quotations from participants who offered particularly articulate, strong, or differing exemplars of each category to best reflect the properties and characteristics of each high-level category and themes. Throughout the interviews and data analysis process, the research group also conducted fortnightly meetings to discuss interview techniques, read transcripts and share analytic interpretations. Team members discussed salient concepts that arose in the interview process and adjusted the grouping of categories and codes. This enhanced the trustworthiness of the data and encouraged the researchers’ reflexivity towards the data.

### Reflexivity

The authors have an external academic perspective on the work of WHO guideline development and implementation, with our understanding mostly guided by official positions stated by WHO [8]. LB has had experience in participating in guideline development groups in WHO, but none of us has direct experience in WHO guideline implementation work in regional and country offices. ZW, QG and LP have diverse clinical backgrounds in pharmacy, nursing and medicine, respectively. During the semi-structured interviews ZW explained he was a doctoral candidate and maintained a “student” position in establishing rapport with the interview participants to encourage exploration of areas of discussion that participants found most representative of their work and that may be previously unknown to the interviewer.

## Results

The characteristics of interview participants are summarised in Table 1. There were 17 interviews with 18 participants (one interview was done with 2 participants as per their request). The topics of the specific guidelines that the participants were involved in have not been provided in order to protect participants’ confidentiality.

**Table 1:** Characteristics of interview participants (*n* = 18)

WHO Offices	No. of Participants Interviewed (***n*** = 18)	No. of Potential Participants Emailed (***n*** = 42)
Headquarters	8	17
Regional	4	8
Country	6	17
**Region of Regional and Country Office Participants**		
*African Region*	*0*	*3*
*Region of the Americas*	*3*	*3*
*South-East Asia Region*	*2*	*5*
*European Region*	*3*	*7*
*Eastern Mediterranean Region*	*0*	*4*
*Western Pacific Region*	*2*	*3*

Reproduced from [23]

### Two models of adaptation

A key finding emerging from the interviews is that the processes to implement and modify guideline recommendations were not uniform; instead we identified a variety of practices described by different participants. We describe two dominate contrasting models of guideline adaptation processes. The models can be viewed as distinct points at either end of a spectrum of possible processes, although they are far from being mutually exclusive.

#### Copy or Customise Model

This pragmatic approach involves either copying or customising WHO guidelines to suit local needs, where the contextualisation of WHO guidelines is done by local health authorities and/or clinicians directly through consultations with WHO staff. Recommendations from guidelines can be used “as is” (i.e., copied) or adapted to the local context (i.e., customized). Selection and adjustment of guideline recommendations are made according to what the implementers deem important, feasible and applicable through the consensus discussions. Those selected recommendations (modified or not) are then defined by the participants as adapted recommendations. The local health authorities and/or stakeholders may take into account the local epidemiology of diseases, health system capacity, availability of interventions, local values and preferences during their deliberations before implementing the adapted guideline recommendations.

#### Capacity Building Model

This model describes WHO building local capacity in evidence synthesis methods and frameworks in order to support local development of a national guideline informed by international guidelines (e.g. WHO guideline but not necessarily limited to WHO guidelines). WHO aims to promote processes similar to those suggested by adaptation frameworks such as the ADAPTE framework [29]. WHO’s role in the capacity building model is to increase capacity in understanding the methods of evidence and guideline review. Local guideline adaptation/development groups can be formed by recruiting and training academics, policy makers, clinicians and stakeholders within the country. Once this methodological expertise is developed WHO assumes that the local guideline adaptors will be able to develop national guidelines informed by WHO or other international guidelines. The recommendations in these local guidelines may be based on their evaluation of the evidence informing these international guidelines, as well as their understanding of their respective local health settings.

The main difference between the two models is the formation of national guideline development groups, which occurs in the Capacity Building Model but not in the Copy or Customise Model. These groups consist of country level stakeholders trained in evidence-based methods of guideline development / adaptation by WHO. They develop national health guidelines based on international guidelines (including WHO guidelines) using processes similar to those in the academic literature [10, 14, 15].

### Model 1: Copy or Customise

Participants discussing this model used the term “guideline adaptation” interchangeably with application, implementation and adoption of guidelines. Ian, a regional officer said “whenever you apply the guidelines you need adaptation by local health system. By adaptation I mean you need the people to apply them.” The exact term used was not important to the participants. The methods they used to contextualise the guidelines also differed greatly from those recommended by G-I-N. Participants understood and assumed that for a guideline to be implemented and used in a setting it had to be contextualized. Participants’ use of the Copy or Customise model was sometimes as simple as adopting some recommendations of WHO guidelines and not others, depending on their respective applicability to the setting in question.

*“The other thing is that we have eradicated [Disease 4] in this country. So, we discontinued the anti- [Disease 4] prophylaxis for pregnant women a long time ago. That is the way that we’re adapting the new guidelines. It depends on the country’s epidemiology and the country data and the situations. Then, gradually, we adapt all - because sometimes although we implement them, we discontinue certain recommendations.”* (Opal, Country Office Staff) The process described by Opal was unstructured. Through consultations with local ministry of health (MOH), parts of WHO recommendations that the local MOH determined to be relevant were implemented, and parts they found irrelevant were left out without the kind of comprehensive process recommended in formal adaptation frameworks like ADAPTE [10]. This informal, flexible model was very efficient in incorporating understandings of the epidemiology and capacity of the local healthcare system into the adapted guidelines.

Examples of different processes of guideline adaptation that fit the Copy or Customise model are summarised in Table 2.

**Table 2:** Examples of guideline adaptation situations in the Copy or Customise model

Situation	Example	Quote
Whole recommendations are adopted	Direct usage of WHO guidelines	*“Often countries that don’t have the resources, don’t have the human or financial resources will just copy and paste our guidelines.”* (Garry, Regional Office Staff)
Parts of recommendations are adopted	Customised to adopt only parts of recommendations AND/OR some recommendations due to medication availability	*“We kind of updated this pocketbook which would be very useful … but we needed the country adaptation meaning the translation and also some of the parts had to be [adjusted], again, because some of the drugs that this group was talking about were missing in the country pharmacy so we had to make an adjustment for that.”* (Judy, Country Office Staff)
Recommendations are customised	Change recommendations due to resource limitations in the context	*“When WHO made the recommendation of … treat all, irrespective of the [Diagnostic Measure 1], that people should be put on treatment … because one is the recommendation to ‘treat all’ but the other is that, okay, if you have limited resources you also need to do prioritisation... so [MOH] didn’t want to move straight to treat all … it was that for all key populations you can start irrespective of [Diagnostic Measure 1].”* (Nicole, Regional Office Staff)

### Relationship with WHO

The adaptation and negotiation process was highly dependent on the specific country’s relationship with WHO. Country staff attributed smooth processes of adaptation to long histories of countries working with WHO. Opal explained,

*“Because we’ve used these WHO guidelines for a long period - 50, 60 years. So, an adaptation of new WHO guidance is not a new thing for the country. It just happens. People know when there’s new evidence or new guidance, we need to adapt it to the country or incorporate it into the existing guidelines. So, things are happening smoothly. But if a country doesn’t have that kind of system, then it will be a little bit challenging.”* (Opal, Country Office Staff)

Participants described how WHO office staff worked closely with the local MOH to understand the local epidemiology and adjust guidelines for disease prevalence, and also considered the capabilities of the local health system and practitioners.

*“[For] that exact practice [to be] be conducted, such as with [WHO guideline 2], you have to transfer that to see what care should be at which level and what other accountable agencies or person to track the action taken under each of the practices, recommended in the guideline. So practical translation into a local context.”* (Ian, Regional Office Staff)

Ian also explained that the care that could be provided at different levels of the health system was dependent on the capabilities available at that level; implementing WHO guidelines that assumed a higher level of expertise than what was available in a local healthcare centre was not practical. Therefore, it was up to the WHO country office staff to “work with technical lead agencies in the country and then review the WHO document … to identify what are the key actions [are appropriate] in where … should be taken by whom” (Ian, Regional Office Staff). In addition to taking the region’s epidemiology and professional workforce into account, the consultations with local governments could also include whether “there is any conflict with existing policies” (Judy, Country Office Staff). All of these factors affected how well the local health authorities might receive WHO guideline recommendations.

In the cases described above, the way a WHO guideline was contextualised in the local setting was directed by WHO regional and country office staff in consultation with local health authorities. It may have included a cut-and-paste process which incorporated some recommendations from the WHO guideline into local standards after considering the local epidemiology, health practitioner/health system capacity and existing local policies.

To accommodate the factors described above, recommendations could also be adjusted until local health authorities and stakeholders reach consensus. This was usually done without a formal evaluation of the evidence and/or other guidelines. The rationale for adopting this process was explained by Opal:

*“Because the WHO has taken all the pain of getting down all the evidence, classifying it, then discussing it among themselves and publishing the guidelines. So, the next step is not challenging the evidence. It’s how to implement this guidance in the country scenario, based on the resources, based on the epidemiology, and based on the systems and other things... I think that the nationals have much more understanding of these kinds of things and the local scenario. So, they are not trying to reinvent the wheel, but they are trying to - how to use that wheel in an effective way in their countries. Whether it’s relevant or not - if relevant, how to use it.”*

**Fig. 1:**
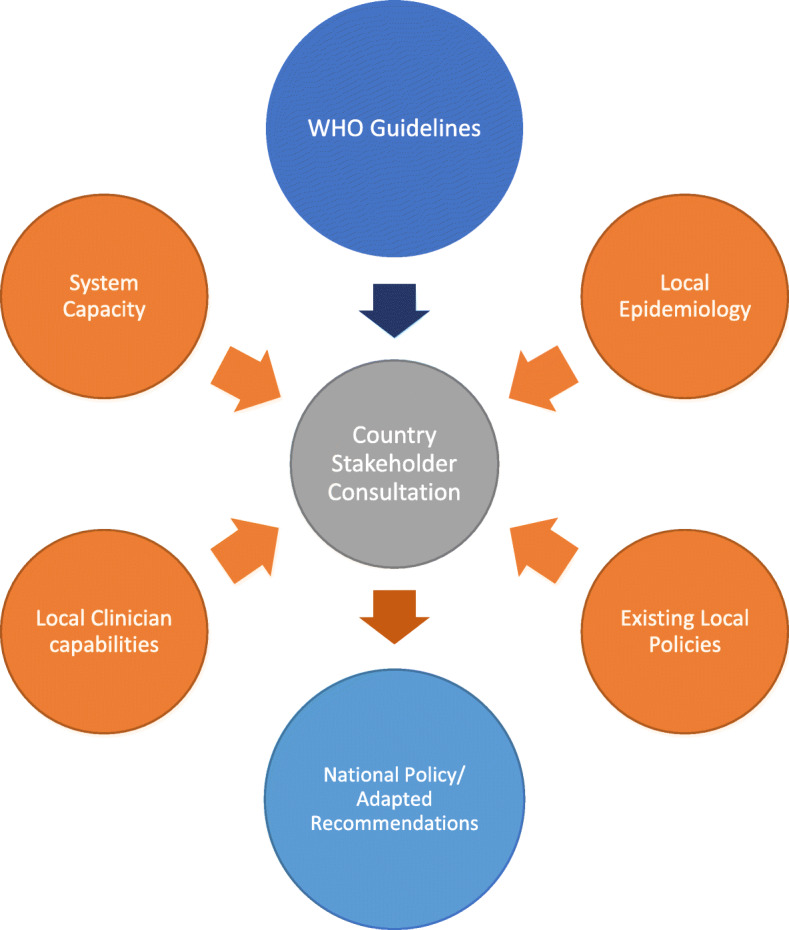
Summary of Model 1: Copy or Customise. Visual representation of the Copy or Customise Model of guideline adaptation

The assumed legitimacy of the evidence informing WHO guidelines was often the major rationale for selecting and slightly adjusting the recommendations in the pragmatic model of guideline adaptation. The process is summarised in Fig. 1 below.

### Model 2: Capacity Building

The Capacity Building Model was primarily used by the Pan American Health Organization (PAHO, the WHO regional office for the Americas). Participants described PAHO’s efforts in the Americas to increase country level capacity in developing national guidelines from the synthesis of evidence as well as existing WHO guidelines, especially in low- and middle-income countries within the Americas region.

In particular, PAHO aimed to increase local capacity and expertise in how to synthesise a body of evidence including the assessment of evidence quality in order to develop evidence-based guidelines (i.e. Grading of Recommendations, Assessment, Development and Evaluation (GRADE) methods).

The various national guideline development programs fostered by PAHO were also encouraged and supported to adapt WHO and/or other guidelines developed using GRADE methods in the process of creating their own guidelines. Participants described this as increasing the countries’ ownership of the guideline process and the guidelines themselves to aid in local acceptance and uptake of the guidelines.

*“A program that supports institutionalisation of the use of evidence in countries, and in particular we support [Lucy, Regional WHO staff] on national guideline programs. So, they use GRADE and they improve their capacities and the standards and development of specific guidelines using GRADE or some of the methods or approaches. So, that’s part of what we do. And in doing that we also have been trying to develop an approach of adaptation of WHO and other guidelines in countries.”* (Kelvin, Regional Office Staff)

According to participants, PAHO saw its work as promoting health through increasing local capacity to develop their own guidelines through GRADE methods. The development could be based on WHO guidelines or other GRADE guidelines, but was essentially driven by local clinicians and academics who had been empowered by WHO and GRADE training. Lucy explained that countries created their “own national guideline even if the base of the evidence is exactly the same, but [they] saw local evidence and [they] followed a whole process, but [they] have the effectiveness and even cost effectiveness in the region; [they] can implement that and adapt that in [their] country.” (Lucy, Regional Office Staff).

The regional officers at PAHO consolidated the methods of GRADE and how to implement them in a handbook for local guideline development programs. They also directly assisted the countries through a “virtual course that [they] use for trying to get people into GRADE” and sometimes the regional officers directly “help [local guideline development programmes] to develop … one or two guidelines so they really understand how this is done” (Lucy, Regional Office staff). In some cases, PAHO might ask a country that was more experienced in the guideline development process to help another country in the region with less experience.

*“For example, we have now formed a collaboration between [country 7] that developed their 23 GRADE guidelines with [Country 8]. They have more GRADE guidelines, and they’re going to provide some [help] from the Ministry of Health national support for developing and supporting [Country 8’s] program, and we are going to support one project with our implementation research project so they can really strengthen the national program in [Country 8].”* (Lucy, Regional Office staff)

**Fig. 2:**
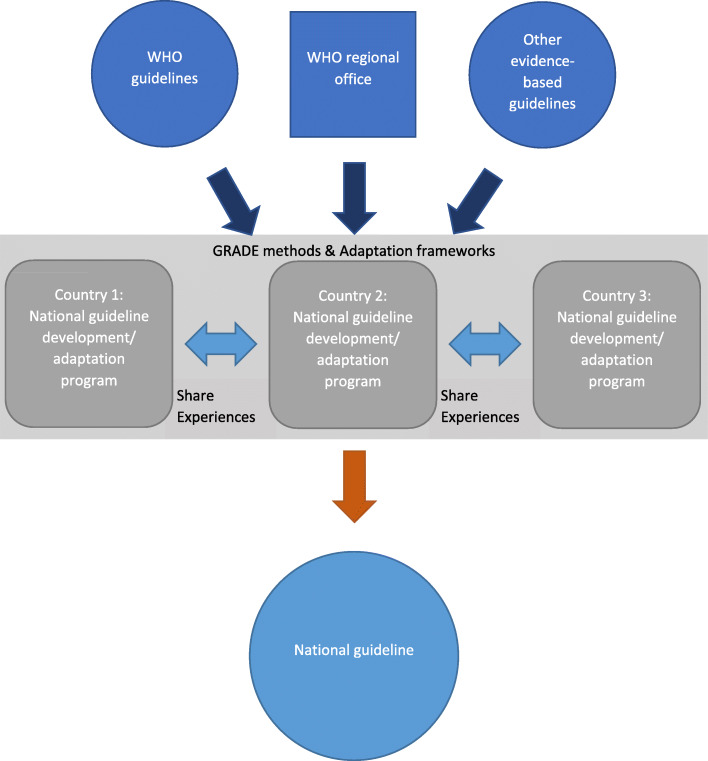
Summary of Model 2: Capacity Building. Visual representation of the Capacity Building Model of guideline adaptation

These capacity building activities allowed WHO regional office staff to work with countries to increase their country level expertise in the process of guideline adaptation. The end goal of this process was for the countries to be able to follow a systematic procedure to adapt or develop their national guidelines in the future without WHO and in extension even help other countries in the region. The features of this model of guideline adaptation are summarised in Fig. 2.

Although primarily used in the PAHO region, the Capacity Building Model was also used in the EURO region. Patrick, a country office staff, explained that in a country in the European region (EURO) they “took a number of global guides of developing guidelines and [they] adjusted it to the [Country 3] system”. Due to the small population of the country they did not have the human resources run large evidence review and guideline development programs like NICE in the UK. So they “needed to think how [they can] adapt the guidelines and … that [they] can develop [their] guidelines, [they] can go through the questions and review the evidence and then come up with a solution which is adapted to the country but also [they needed] to look to the broader adaptation”. They developed a protocol for developing national guidelines modelled after the WHO guideline review committee’s methods to have a more systematic way for developing and adapting guidelines. This is a model of guideline development similar to the capacity building model, but it was initiated by the country’s health authorities themselves rather than being an initiative by the regional office.

## Discussion

The contrasting models of guideline adaptation encountered in the interviews show the differing processes employed by WHO staff in different regions in the world. One is a Copy or Customise model of adaptation through consultation with local health authorities and stakeholders. This model takes parts of WHO guidelines and applies them as is or in modified form in-county, while taking into account the local epidemiology, system capacity and existing policies. The other model is based on capacity building where WHO regional offices help build and support national guideline development and adaptation programs so national guidelines can be developed based on WHO or other evidence-based guidelines using thorough framework for evidence evaluation to inform the strength of recommendations in the local setting. The actual processes of guideline adaptation revealed by our interviews existed along a spectrum between these 2 models. A better understanding and appreciation of both of these models of guidelines adaptation would be useful to guide adaptors and implementers of guidelines in designing their operations in the future according to their specific situations.

The Copy or Customise model may have been derived from situational characteristics in certain health systems and settings. Local decision-making agencies (e.g. MOH) have the ultimate authority to choose which recommendations are to be implemented in the country based on their understanding of the local conditions. This process seems to have been built on and facilitated by WHO officers’ interactions with each countries’ MOH. Compared to the capacity building model, it requires less human and fiscal resources as the implementation of guidelines directly follow, or is completely integrated with adaptation.

The Capacity Building model proposed by PAHO places emphasis on the use of evidence-based guideline development/adaptation methods to enable local academics and guideline development groups to create their own national guideline based on available WHO and other high-quality evidence-based guidelines. PAHO has since published a guideline for their region promoting adaptation through this process [30].

### Models on a spectrum

Guideline adaptation should be viewed as a spectrum with each of the two models described in this study sitting at either end. These models are far from mutually exclusive. The WHO Eastern Mediterranean region (EMRO) has recently published a resolution on “Developing national institutional capacity for evidence-informed policy-making for health” [31] and promotes different degrees of use of evidence-based methods in guideline adaptation depending on the context of implementation.

The WHO Handbook for Guideline Development currently contains little guidance on the contextualisation and adaptation of guidelines in the settings where they will be implemented. It describes guideline adaptation in terms of the ADAPTE framework and the Guidelines International Network [8]. The process of guideline adaptation proposed by the ADAPTE framework is idealistic and not reflective of most cases where WHO guidelines are modified to the local context. The adaptation frameworks in the literature, the WHO Handbook for Guideline Development and indeed the evidence-based medicine paradigm overtly favours processes similar to the Capacity Building Model.

#### Value of the copy or customise model

It would be useful for descriptions of the adaptation process in the WHO Handbook for Guideline Development to include the various models of how contextualisation could potentially occur. For example, guideline adaptors could consider a potential negotiation process with local health authorities as described for the Copy or Customise Model. In addition, the handbook could suggest adjusting the adaptation approach depending on the local epidemiology, health system capacity, existing health polices, and the relationship between the local setting with WHO and WHO. As these contextual factors may limit the ability for guideline adaptors to strictly follow the Capacity Building Model.

#### Expansion and clarification of the process of adaptation

An expansion of the academic definition for the process of guideline adaptation is needed to recognise the spectrum of models. Various groups (e.g. GRADE and ADAPTE working group) have sought to define and promote a version of guideline adaptation based on their own frameworks [10, 14, 15]. The ideal process described by the adaptation frameworks should be recommended when expertise and resources are available. But when those conditions cannot be met, systematic methods for taking local conditions (e.g. epidemiology, capacity) into account can be derived from WHO experiences (i.e. the Copy or Customise model). In any setting where guidelines created in another context are to be implemented (e.g. regional, national or subnational level), a negotiation process needs to take place to contextualise the guideline (with or without the formation of an official guideline adaptation group/program).

If the definition of guideline adaptation in the WHO Handbook for Guideline Development was to expand to include and recognise the processes described by the participants of this study that are already taking place, it would be a better guide for guideline implementers to contextualise guidelines depending on the setting of adaptation. A clarification of the terminology around “guideline adaptation” would also be helpful given the widespread interchangeable use of terms encountered in the interviews. Further research into and refinement of the Copy or Customise model could be useful to guide future guideline adaptors and implementers through various barriers that may arise.

### Major contributions

To the best of our knowledge, this study is the first to focus on a range of experiences of WHO regional and country level officers in guideline adaptation and implementation. Previous studies have been limited to isolated case studies of guideline implementation measuring the successful uptake of recommendations [17–19]. Previous studies also provided data on the barriers and facilitators of guideline implementation, but did not examine the experiences of WHO staff who have adapted and implemented numerous guidelines across diverse regions in the world. By analysing the range of experiences explored in our interviews, we were able to formulate the models of guideline adaptation described in this paper.

The results of this study also build upon the current academic understanding of guideline adaptation [10, 12–15]. Studies that describe guideline adaptation frameworks often contain examples of large projects in a particular country context (e.g. Norway [13], Saudi Arabia [15]). The processes promoted by these frameworks are similar to those we describe for the Capacity Building Model, but our results show that this is only a part of the spectrum of models that are used for guideline adaptation. Recognising that, in reality, processes of guideline adaptation exists on a spectrum (from the Capacity Building Model to the Copy and Customize Model), is an important addition to future guidance on guideline implementation in the future.

### Limitations

Consistent with qualitative methodology [32, 33], this was a convenience sample of participants, within a purposively sampled recruitment frame. Thus, findings aim to show the range of possible experiences among WHO guideline implementers. Particularly, in our sample, perspectives from the African and Eastern Mediterranean regions were not represented due to recruitment limitations. There may be additional issues particularly relevant to these regions that we have missed. Future studies could focus on the experience of guideline adaptation and implementation in these regions. However, as the first in-depth understanding of the process of WHO guideline implementation, this analysis offers a unique contribution to the understanding of how evidence-based interventions and policies are actually put into practice. By exploring the differences between the regions, future studies can also reflect on the inherent differences in capacities at the regional and country offices and what do they need in order to optimally support Member States.

### Implications

The adoption of evidence-based methods of guideline adaptation/development is a long process that is highly dependent on both the availability of methodologists in the regional offices and expertise within the country where capacity building takes place, as well as other resources. In settings where these conditions cannot be met, it is difficult to initiate this process. However, other models of guideline adaptation could be practical for different regions of the world. Understanding of these differences would be important to shape the work of WHO in the guideline implementation process. To increase the uptake of WHO guidelines and recommendations different types of support are needed for the spectrum of models of guideline adaptation.

#### Copy or Customise Model

For the Copy or Customise model to more effectively guide local country health policy, WHO guideline developers can include considerations for the potential “cut and paste” or minor modification of recommendations within a guideline by local health authorities during the implementation process. In cases where the most evidence-based option is not available, guideline recommendations for first line and second line intervention options may be helpful for local implementers to select recommendations to implement. In addition, suggestions for the modifiability of recommendations in guidelines would give local implementer a guide to the essentiality of a recommendation (e.g. labelling recommendations as “essential, non-modifiable” vs. “adjustable according to local capacity”). Increasing WHO support from each level of offices (HQ, regional and country) in the negotiation process with local health authorities and stakeholders would be able to clarify each of the factors summarised above when necessary.

This stratification of guideline recommendations has been experimented with by the US National Comprehensive Cancer Network (NCCN) in their NCCN Framework for Resource Stratification of NCCN Guidelines [34]. Guidelines could have separate recommendations labelled for basic level of resources and for enhanced level of resources. This clarifies the importance and resource requirements of different recommendations of the adaptors, implementers and end-users of a guideline. It would also make the Copy or Customise model more efficient and adapted recommendations more evidence-based (as the evidence review for the recommendation options has been performed by the original guideline developers).

#### Capacity Building Model

For settings where the Capacity Building Model is prevalent, increasing the availability of methodologists and training in evidence evaluation and guideline development would be essential to build and support local guideline development/adaptation programs. This could be closely linked to WHO fostering collaboration between countries to share their experiences and expertise in building local guideline development/adaptation capacity. Countries in a similar region can share their valuable experiences in building a guideline development/adaptation program with less experienced countries. This expands the base of methodology experts, and the practical experiences guideline developers from the same region can share may be even more poignant than assistance offered by methodologists from other settings. From WHO guideline developers’ perspective, a higher availability of evidence to decision tables from the original WHO guidelines would fast-track local processes.

In addition, the GRADE methodology used by WHO and encouraged by PAHO for national guideline development programs has a focus on clinical practise questions (comparing interventions for specific outcomes). The rating of certainty of evidence is skewed in favour of randomised control trials (RCTs), with RCTs at the high end of the scale for the quality of evidence, while observational studies usually start at the low grade due to residual confounding [35]. For public health guidelines where observational studies are the majority of the studies available, a strict application of the GRADE methods may result in a large amount of weak/conditional recommendations [36]. In public health questions, where RCTs are not available or not the most common study design (e.g. due to ethics, impracticality, and/or cost), GRADE methods should be modified to suit the guideline adaptors’ and policy makers’ purposes [37]. Other guideline adaptation and implementation frameworks can also be drawn upon to refine this model. For example, the CAN-IMPLEMENT framework [38, 39] guides users through the implementation process on a digital platform. This feature can encourage guideline adaptors and policy makers to plan implementation of their guidelines during and after the adaptation process.

## Conclusions

The adaptation of WHO guidelines has been understood and practiced differently in different regions across the world. This paper highlights two contrasting models of guideline adaptation.

We outline the different kinds of support from guideline developers and WHO for these contrasting models of guideline adaptation to improve their effectiveness and efficiency in the future. Understanding the differences between the models would be important to shape the work of WHO in supporting the guideline adaptation and implementation process.

We also suggest an expansion and clarification of the process of guideline adaptation in WHO and academia in general. A decision aid for various models of guideline adaptation would be helpful for guideline adaptors and implementers to decide on the appropriate course of action according to the circumstances they encounter and identify potential paths for improvement.

## Supplementary Information

**Additional file 1.** Interview Guide. The interview guide for key areas of discussion used in the interviews.

## Data Availability

The datasets generated and/or analysed during the current study are not publicly available in order to protect participant confidentiality.

## Abbreviations

EtD: Evidence to decision tables
EURO: WHO Regional Office for Europe
GRADE: Grading of Recommendations, Assessment, Development and Evaluation
GRC: WHO Guideline Review Committee
MOH: Ministry of Health
NHMRC: National Health and Medical Research Council
NICE: The National Institute for Health and Care Excellence
NIH: National Institutes of Health
PAHO: Pan American Health Organization
WHO: World Health Organization
WPRO: WHO Regional Office for the Western Pacific
